# Unexpected, but consistent and pre-registered: Experimental evidence on interview language and Latino views of COVID-19

**DOI:** 10.1177/20531680231168736

**Published:** 2023-04-19

**Authors:** Efrén Pérez, Jessica HyunJeong Lee, Ana L Oaxaca Carrasco, Cole Matthews, Madison Ritsema

**Affiliations:** 1 Departments of Political Science & Psychology, UCLA, Los Angeles, CA, USA; 2 Department of Political Science, UCLA, Los Angeles, CA, USA; 3 Department of Psychology, UCLA, Los Angeles, CA, USA

**Keywords:** COVID-19, Latino public opinion, interview language

## Abstract

Much uncertainty remains about effective messaging to boost public support for COVID-19 mitigation efforts, especially among people of color. We investigate the relationship between interview language and expressed support for COVID-19 public health protocols among Latinos: America’s largest ethnic group. Prior work establishes that interview language shapes opinions by *cognitively* structuring which considerations people use to express attitudes. Yet other work suggests interview language shapes opinions by activating specific *cultural* norms associated with a tongue. We predicted that interviewing in Spanish (versus English) would boost support for COVID-19 protocols by activating *pro-social norms* known to be strongly associated with that language. We uncover null support for this prediction in a pre-registered experiment on bilingual Latino adults (*N* = 1645). Instead, we find that Latinos assigned to interview in Spanish report weaker support for COVID-19 protocols, regardless of which cultural norms are primed. We discuss implications for COVID-19 attitudes in linguistically diverse polities.

A lasting U.S. effect of the COVID-19 pandemic is the stark racial/ethnic imbalance in terms who gets sick and perishes from the novel coronavirus. Public health data reveal substantially higher mortality rates for people of color (PoC), especially Latinos and African Americans, than non-Hispanic Whites (CDC Cases et al., 2020). These disparities are traced to greater exposure to the coronavirus that many PoC face as front-line workers in service industries (Asfaw, 2021), plus continued resistance to (booster) vaccinations and other mitigation efforts (Gaffney et al., 2022). One cost-effective tool to soften opposition to anti-COVID-19 efforts is public communications that increase support for them. Yet fledgling literature on mass opinion toward COVID-19 mitigation measures centers primarily on non-Hispanic Whites, while revealing few effective messages that boost support for COVID-19 health protocols among them (Gadarian et al., 2022). We contribute here by testing the effects of interview language on personal support for COVID-19 protocols among Latino adults: America’s largest ethnically minoritized group.

Growing research suggests that interview language influences the opinions that Latinos and other linguistically minoritized groups hold (Pérez and Tavits, 2022; Flores and Coppock, 2018; Welch et al., 1973). One framework explains these language-opinion effects as emerging from the connections that exist between different languages and varied content in memory. This mechanism suggests that specific languages provide easier access to some mental considerations, thus shaping the sample of contents that individuals use to express opinions (Zaller, 1992). For example, across national surveys of Latino adults, Lee and Pérez (2014) find that interviewing in English (versus Spanish) yields stronger views and knowledge about U.S. politics, net of covariates. Experimental evidence suggests these patterns arise because different languages stimulate specific contents in memory (Pérez and Tavits, 2022). Consider Latino attitudes toward American politics, which are produced via considerations that are primarily (although not exclusively) learned in English, such as U.S. civic facts and symbols. Given this link between language and content in memory, Latinos assigned to interview in English (versus Spanish) report stronger American and Latino identities (two categories learned in the U.S.), but weaker identities as Mexicans, Cubans, Puerto Ricans, etc., (i.e., categories native to Latin America) (Pérez, 2016). These patterns also appear in (non-) U.S. settings (Pérez and Tavits, 2022).

With roots in the psychology of survey response (Tourangeau et al., 2000), scholars dub this a *cognitive* account of language-opinion effects. This theory predicts that Latinos who interview in Spanish (versus English) will report weaker support for COVID-19 public health protocols because the information needed to evaluate these measures is overwhelmingly disseminated in English (cf. Marian and Neisser, 2000; Pérez, 2016). Indeed, available research reveals a strong asymmetry in the supply of public information about COVID-19, with Spanish language media (e.g., Univisión and Telemundo) offering less overall volume and more disinformation than their English counterparts (Mochkofsky, 2022; The State of Latino News Media, 2019; Pew Research Center, 2021). In our theory section, we return to this informational assumption behind the *cognitive* account of language effects and support it empirically.

In contrast to this *cognitive* framework, some social psychologists propose a *cultural* explanation for language effects (Markus et al., 1991; Triandis, 1993; Trafimow et al., 1997). These scholars reason that people inhabit societies deeply characterized by *individualist* versus *collectivist* cultures. *Individualist* cultures are characterized by independence and affirm a person’s unique goals and aspirations. In contrast, *collectivist* cultures are characterized by *inter*dependence between individuals, where a person’s needs are understood only with respect to the larger communities they belong to. Thus, *individualist* cultures privilege the self over the collective, while *collectivist* cultures are pro-social, prioritizing larger communities over the unique person.

These broad cultural differences manifest through the norms and values that people express in specific situations. Since language is assumed to operate as a reliable indicator of these broad cultural nuances, the degree to which *collectivist* or *individualist* norms are expressed in a particular setting will depend on interview language. Indeed, previous work establishes that the degree to which cultural contents are encoded in memory varies reliably by the language one uses in a situation (Marian and Neisser, 2000; Marian and Kaushanskaya, 2007), with previous scholarship establishing that Spanish is more strongly associated with *collectivism*, while English is more strongly associated with *individualism* (Shkodriani and Gibbons, 1995; Gabrielidis et al., 1997).

This research often uses interview language to manipulate cultural differences between people (Hong et al., 2000). The reasoning is that language is an indicator of cultural content in people’s long-term memory (Collins and Loftus, 1975). Thus, the very act of using a specific language (e.g., reading, speaking, and writing) is believed to activate cultural norms and make them mentally accessible when forming judgments or opinions. Such activation typically occurs through *lexical* or *grammatical* features of a language (Perez and Tavits, 2022). For example, in studies of Mandarin/English bilinguals, participants who interview in Mandarin (associated with a *collectivist* culture) report reliably different self-concepts, emotions, and personality profiles than participants who interview in English (associated with an *individualist* culture) (Bond, 1983; Matsumoto and Assar, 1992; Chen et al., 2014; Trafimow et al., 1997). These effects depend on the cultural norms associated with a specific language, rather than just the interview language alone. That is, language-opinion effects occur only when the “right” cultural norms are activated. Applied to Latinos’ support for COVID-19 mitigation efforts, this framework implies they will report *more* support for these health measures when they interview in Spanish (versus English) because this language is strongly linked to a pro-social, *collectivist* culture. Hence, interviewing in Spanish should activate pro-social norms, which directly increases support for COVID-19 mitigation measures that aim to stall this disease’s spread.

We experimentally evaluate these perspectives in the context of Latino opinion toward COVID-19 public health protocols. As the more compact account, we treat the *cognitive* perspective on language effects as our null hypothesis (H0). Here, interviewing in Spanish (relative to English) should dampen Latino support for COVID-19 public health protocols because considerations necessary to express an opinion are largely ones encountered in English (Pérez, 2016; Pérez and Tavits, 2022: Chapter 6), as depicted by Figure 1. Per Zaller (1992), Tourangeau et al. (2000), and Schwarz (2007), considerations are the information that people learn about and store in memory—in this case, about COVID-19—that allow them to form and express opinions. This prediction means language-opinion effects in this domain should emerge without the additional help of cultural norms. In turn, our rival hypothesis (H1) is that interviewing in Spanish (versus English) produces *greater* support for COVID-19 public health protocols in light of *collectivist* norms, which are pro-social in nature. In other words, the activation of *collectivist* norms, via interviewing in Spanish, should increase support for COVID-19 policies aimed at mitigating the negative effects of the pandemic on the collective public.

**Figure 1.**
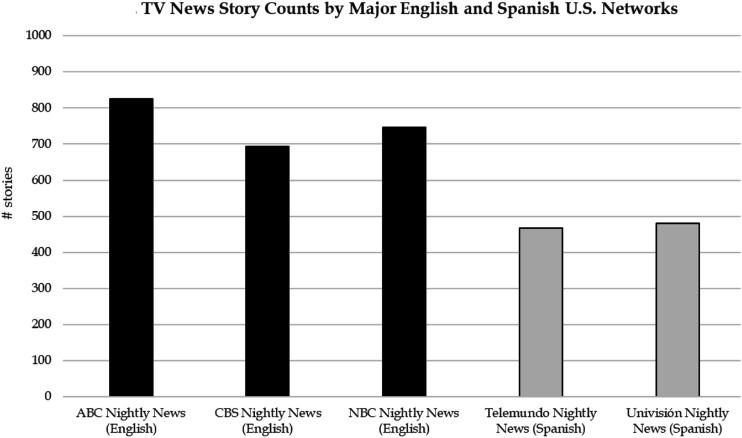
TV news story counts by major English and Spanish U.S. networks (source: authors' analysis).

We assess these pre-registered hypotheses with a 2 (interview language: English and Spanish) x 2 (norms: collectivist, individualist) experiment on 1645 U.S. Latino adults. After appraising their baseline attitudes toward four COVID-19 mitigation measures, participants (Ps) were assigned to interview in English or Spanish (factor 1). We crossed this manipulation with another treatment providing information about COVID-19 health protocols using *individualist* or *collectivist* language (factor 2). This design produces four conditions that let us observe whether interview language effects vary by the specific norms that are primed. Contrary to a *cultural* account of language effects, we find that Latino adults assigned to interview in Spanish express significantly weaker support for COVID-19 public health measures when primed with *collectivist* norms (−0.051 percentage points, SE = 0.020) and *individualist* norms (−0.063 percentage points, SE = 0.020). We also find that support for COVID-19 mitigation efforts significantly declines, relative to baseline, among Ps who were assigned to interview in Spanish and faced *collectivist* norms (−0.016 percentage points, SE = 0.009). We discuss our results’ implications for COVID-19 public health messaging and consider possible synergies between the evaluated theoretical accounts.

## Theories and hypotheses

The *cognitive* account of language effects draws on a *belief-sampling* mechanism (Zaller, 1992; Tourangeau et al., 2000). It reasons that most people hardly have any ready-made opinions to report about public affairs. What they have instead are *considerations*—beliefs, values, knowledge, and other orientations—which are cobbled together to express an opinion. When individuals encounter a survey question, various mental contents in memory are triggered. This leads to a process of s*preading activation*, where the initial stimulation of content triggers additional related considerations. *Belief-sampling* implies that the ultimate batch of considerations that are dredged from memory will be highly sensitive to situational features in a survey context (Tourangeau et al., 2000), which includes the language one interviews in. *Belief-sampling* also implies that considerations in memory are learned in specific languages (Marian and Neisser, 2000). Consequently, the sample of considerations recruited from memory will vary by interview language, and this nuance should yield reliable opinion differences.

In terms of U.S. attitudes toward COVID-19 mitigation measures, this framework assumes that an overwhelming number of considerations are disseminated in English. We support this assumption in three ways. First, Figure 1 displays the number of news segments about COVID-19 that were broadcast in nightly newscasts (Monday–Friday) by five major television networks: ABC (English), CBS (English), NBC (English), Telemundo (Spanish), and Univisión (Spanish). These data are from the *Internet Archive -TV News*, an online repository of TV broadcasts from 2009 to the present. The period here is from 1 January 2020 through 30 June 2022. While all five networks broadcast a sizeable number of stories about COVID-19, there is a significant asymmetry in the number of stories from English networks (M_English_ = 754.33) versus Spanish ones (M_Spanish_ = 474.50) (M_English_ – M_Spanish_ = 279.83, t = 5.62, *p* < 0.011, two-tailed).

Second, as a public source of credible information about COVID-19, the Centers for Disease Control and Prevention (CDC) maintains an active online presence through social media, including Facebook and Twitter. These efforts include social media feeds that are targeted at English- and Spanish-speaking individuals. Table 1 shows that, as of 10 January 2022, the top 10 most frequented Facebook and Twitter feeds sponsored by the CDC are primarily English-based, with Spanish-based feeds attracting less online traffic.

**Table 1.** Top social media feeds sponsored by the CDC.

Facebook pages	Twitter handles
1. CDC (English)	1. CDC gov (English)
2. CDC Global (English)	2. CDC Emergency Preparedness (English)
3. Nat’l Inst., Occupational Safety (English)	3. CDC Flu (English)
4. CDC Emergency Preparedness (English)	4. CDC eHealth (English)
5. CDC en Español (Spanish)	5. CDC Director (English)
6. CDC Tobacco Free (English)	6. Nat’l Inst., Occupational Safety (English)
7. Start Talking Stop HIV (English)	7. CDC Global (English)
8. Millions Hearts (English)	8. CDC Cancer (English)
9. CDC Traveler’s Health	9. CDC Español (Spanish)
10. Veto Violence	10. CDC HIV (English)

Finally, journalists (Mochkofsky, 2022; Guyn and Murphy, 2021), media watchdog organizations (Longoria et al., 2021), and social scientists (Velez et al., 2022) have brought systematic attention to the prevalence of misinformation about COVID-19, especially in online Spanish-language platforms that are lightly monitored and moderated. As Mochkofsky (2022: 1) explains, “many Spanish-language social-media pages and groups are ‘cesspools’ where disinformation [about COVID-19] thrives uncontested.” Thus, part of the asymmetry in information available to Latinos likely consists of differences in the prevalence of misinformation, with relatively more misinformation about COVID-19 circulating in Spanish.

These three trends align with our theoretic assumption about linguistic asymmetries in the volume of COVID-19 information available to Latinos. At its core, this assumption implies that the samples of considerations related to COVID-19 that Latinos possess vary significantly by language (Spanish and English). This reasoning yields our null hypothesis (H0), which is rooted in a *cognitive* account of language effects: Latinos who interview in Spanish (versus English) will report weaker support for COVID-19 protocols. By this view, Latinos who are called to express their opinions in Spanish (versus English) will draw on a sample of considerations about the *coronavirus* that, in expectation, is less extensive and more suffused with misinformation.

In contrast, our alternative hypothesis draws on a *cultural* account of language effects. An attraction of this framework, in the context of COVID-19, is its emphasis on the activation of *pro-social norms* that can incentivize individual compliance with health protocols that benefit a collective (i.e., mass public). Although this *cultural* framework also relies on *belief-sampling*, its distinction is the stimulation of specific cultural norms, whose type depends on interview language (Trafimow et al., 1997; Hong et al., 2000). If *collectivist* cultures (vs. *individualist* cultures) privilege strong *inter*dependence between people, where a person’s needs are understood only with respect to the larger communities they belong to, then interviewing in a language associated with a *collectivist* culture should activate *pro-social norms* that prioritize larger communities over the unique person (Bond, 1983; Trafimow et al., 1997; Chen et al., 2014). In the case of U.S. opinion toward COVID-19, this perspective implies that a language’s association with a specific culture is likely to activate particular norms that influence one’s attitudes. In the realm of public health, adherence to mitigation measures against COVID-19 is quite variable, with some individuals viewing it as a personal matter and others viewing it as a social obligation (Hearne and Niño, 2022). Thus, we hypothesize (H1) that Latino support for COVID-19 protocols will be stronger among individuals who interview in Spanish because this language is more tightly linked to a *collectivist* culture that enshrines *pro-social norms* (Triandis, 1993).

## Experimental design

We test our predictions with a 2 (interview language: English and Spanish) x 2 (norms: collectivist, individualist) experiment on *N* = 1645 bilingual Latino adults. This pre-registered study used a pre-/post-test design, which lets us appraise language effects on *levels* of and *changes* in opinion toward COVID-19 public health protocols (see SI.4 for pre-registration). Through LUCID’s^™^ online platform, we screened Latino adults for self-reported competence in English and Spanish, which aligns with prior work (Pérez and Tavits, 2022). We asked adults “How well do you read and understand English?,” using a scale ranging from 1) *I do not read and understand English very well* to 4) *I am completely fluent in English*. A similar item was completed in Spanish. Individuals qualified for participation if they reported they were *very* or *completely* fluent in each tongue. Prior work indicates that self-reported reading fluency is highly correlated with a) reading comprehension, b) speaking ability, and c) writing ability in a specific language (Pérez and Tavits, 2022).

After confirming their *Latino* ethnicity, participants (Ps) were informed that based on their reported fluency in English and Spanish, they would continue the interview in one of those languages. On a random basis, Ps were assigned to interview in English or Spanish. After this manipulation, Ps answered a brief battery of demographic items (i.e., age, gender, education, and nativity) (SI.1–SI.2 report sample composition and balance tests). Ps then completed four baseline measures of attitudes toward COVID-19 health protocols, with each item answered on a scale from 1-*strongly oppose* to 7-*strongly support*:

1) The CDC is thinking about issuing a new national mask mandate to slow the transmission of COVID-19. What is your opinion about this proposed measure?
2) The CDC is also considering limiting restaurants and stadiums to 50% capacity to slow the transmission of COVID-19. What is your opinion about this proposal?
3) The CDC is thinking about making it a requirement to show proof of vaccination for activities in public spaces, such as restaurants and stadiums. What is your opinion about this proposal?
4) The CDC is also considering a mandatory quarantine of 7 days for domestic and international travelers if one is infected with COVID-19. What is your opinion about this proposal?

We combine these items into a summated index (α_pre_ = 0.875), which we rescale from 0 to 1. Importantly, the average level of support falls just above the midpoint of this scale (M = 0.597, SD = 0.280), with substantial variation around its central tendency. This provides some reassurance that any treatment effects (or lack thereof) are an unlikely function of ceiling or floor effects due to “pandemic fatigue” stemming from possible over-saturation of COVID-related public messaging.

After assessing these pre-treatment attitudes, Ps were assigned to read a message about compliance with COVID-19 public health protocols. These messages were attributed to the CDC and used the same core message and graphic (see SI.3). Each message primed *individualist* or *collectivist* norms, resulting in four total experimental cells: *English interview-individualist norms*, *English interview-collectivist norms*, *Spanish interview-individualist norms*, and *Spanish interview-collectivist norms*. For instance, in the *English-individualist* condition, Ps were exposed to a CDC communiqué titled: “Amid a plateau in COVID-19 vaccinations, the Centers for Disease Control and Prevention (CDC) reconsiders a new mask mandate and stronger efforts to boost vaccination rates *among individuals”* (emphasis added). Each communiqué was accompanied by a CDC graph underlining the thrust of each message in each language. We did this to facilitate the processing of our overall treatment. This focus on *individualism* was affirmed throughout the news brief with words focused on *individual* and *personal sacrifices*, and explicitly recommended adhering to public health protocols by noting that “You should do it for yourself. You should do it for your own health.” We primed *collectivist* norms by replacing these words and phrases with pro-social analogs, including *communities* and *collective sacrifices*, as well as an exhortation to follow public health protocols by doing this “for our communities” and “for our communities’ health.”^1^ Our manipulation here is a conceptual extension of prior work by priming cultural norms via text and visuals.^2^

After our treatment, participants completed a manipulation check, consisting of a true/false item verifying the content of an assigned article (“The article I read was focused on a new CDC consideration to reinstate a national mask mandate and increase efforts to increase vaccinations and boosters”). One hundred thirty-two participants (*n* = 132) failed this check, which is only about 7% of the total number of participants we initially recruited. Per our pre-registration, our analytic sample consists of the grand majority of individuals who passed this check (93%). Respondents who failed this check were terminated at that point in the survey. We did this to rule out respondent inattention by design, given the processing effort that our manipulation requires. Although there is a risk of inducing post-treatment bias via this approach (Montgomery et al., 2018), this tradeoff is not an issue in our study, since we find balance on the pre-treatment variables we collected (see SI.2).

Following our manipulation check, Ps again answered our baseline opinion measures (α_post_ = 0.891; M = 0.603, SD = 0.281). This high degree of measurement reliability further reassures us that any subsequent results are not driven by respondents who misreport their degree of bilingualism. If that were the case, we should have observed here a low degree of measurement reliability (i.e., high measurement error), yet we observe a robust degree of precision in these measures.

We use our COVID-19 opinion measures to estimate two models. In an unadjusted model, we estimate the effect of our treatments on our post-treatment outcome, *support for COVID-19 protocols*. This model evaluates whether our treatment(s) reliably impact levels of *support for COVID-19 protocols* in any way. In an adjusted model, we then estimate the same effects but include pre-treatment opinions about COVID-19 measures as a covariate. This lets us evaluate whether any treatments directionally *change* opinions, rather than simply affecting their reported levels. In our analyses, all variables are re-scaled to a 0–1 interval, allowing us to interpret all coefficients as percentage-point shifts. Unless otherwise indicated, all reported *p*-values are two-tailed.

## Results

Table 2 reports our (un)adjusted model. Here, Ps who interviewed in English and received *individualist norms* serve as controls. We find clear evidence against (H1). All reliable treatment effects emerge among Ps assigned to interview in Spanish. For example, in comparison to Ps who interviewed in English and received *individualist* norms, those who interviewed in Spanish and received *individualist* norms are about six percentage points less supportive of COVID-19 protocols (−0.063, SE = 0.020, *p* < 0.001). Similarly, compared to Ps who interviewed in English and received *individualist* norms, those who interviewed in Spanish and received *collectivist* norms are five percentage points less supportive of the same COVID-19 protocols (−0.052, SE = 0.020, *p* < 0.008). In sharp contrast, Ps who interviewed in English and encountered *collectivist* norms were negligibly more supportive of COVID-19 protocols (0.0002, SE = 0.019, *p* < 0.993).

**Table 2.** Estimated treatment effects of interview language on Latino support for COVID-19 public health protocols (unadjusted and adjusted).

	Support COVID-19 protocols (unadjusted)	Support COVID-19 protocols (adjusted)
English interview-*collectivist norms*	0.0002 (0.019)	−0.008 (0.009)
Spanish interview-*individualist norms*	−0.063** (0.020)	−0.005 (0.009)
Spanish interview-*collectivist norms*	−0.052** (0.020)	−0.016* (0.009)
Support COVID-19 protocols (pre-treatment)	—	0.897* (0.011)
Constant	0.631** (0.014)	0.075** (0.009)
Root MSE	0.280	0.126
N	1645	1645

*Note:* **p < 0.05, *p < 0.10, two-tailed.

These results contradict the hypothesis (H1) that cultural norms will condition the impact of interview language on support for COVID-19 protocols. Instead, the findings align better with (H0), where language shapes the mental considerations that are retrieved from memory, without the added influence of cultural norms. Of course, one might reasonably wonder whether our English-based treatments were interpreted as targeting *Americans* in general, while our Spanish-based treatments were perceived as Latino-specific. We think it is unlikely given the design of our treatments. Our manipulation references *Americans (estadounidenses)* only once, with this mention appearing in each treatment article. Thus, this is a feature that is held constant across all treatment conditions (including the control group). This means the only variation in our treatments comes from the language that is used and whether it primes *individualist* or *collectivist* norms. Additionally, if our treatments had operated in this alternative way, at minimum, we would expect a reliable and positive effect from our *English-collectivist norms* treatment, yet we observe no substantively or statistically meaningful effect from this condition.

To further probe our findings, we re-estimate our observed treatment effects and adjust them for pre-treatment levels of support for COVID-19 protocols. This allows us to dampen sampling variability, while appraising whether any of our treatments directionally change people’s support for these COVID-19 protocols from baseline.

This analysis shows that the correlation between pre- and post-treatment levels of support for COVID-19 protocols is high, as expected, but it is not unity (0.897, SE = 0.011, *p* < 0.001). Therefore, although pre-treatment support for COVID-19 protocols is strongly associated with post-treatment support of these initiatives, one of our treatments changes these opinions in a negative direction. Specifically, Ps who interviewed in Spanish and received *collectivist* norms are nearly two percentage points *less* supportive of COVID-19 public health protocols (−0.016, SE .009, one-sided *p* < 0.039).

Per our theorizing, this is the condition where one should observe a stronger effect in a positive direction, given the stronger fit between a person’s tongue (Spanish) and the social norms they are associated with (*collectivist*). Yet we find the opposite pattern.

## Implications

We surmised that language could increase support for COVID-19 health protocols by activating *pro-social norms* that encourage individual engagement with efforts that affect larger communities. Our large-scale experiment revealed that, in comparison to Latinos who were assigned to interview in English, those who were assigned to interview in Spanish reported weaker support for public measures to mitigate COVID-19’s spread, a pattern that more neatly aligns with a *cognitive* account of language effects. What does this mean in theoretic terms?

While it may seem intuitive to conclude that our results confirm the *cognitive* account of language effects as better than the *cultural* account, we caution against this premature conclusion. First, in the null hypothesis significance testing (NHST) framework that we (and many political scientists) used, a researcher is hard-pressed to ever prove the null. Yes, our evidence here does not support a more involved *cultural* account of language effects, but support for the more compact *cognitive* is one that occurs by default. We stress this point given the timing of our experiment, which is about 2 years after the onset of the COVID-19 pandemic. Although we showed that Latino participants’ baseline opinions concerning public health protocols around COVID-19 are not prone to ceiling or floor effects, it is entirely possible that “pandemic fatigue” could have influenced our null results in ways that our specific research design cannot fully accommodate (e.g., pre-treatment effects, pandemic fatigue as a treatment moderator).

Discarding the *cultural* account is also unwarranted considering that our results—like any experimental findings—are dependent on the domain we studied (COVID-19 opinions), our treatments’ operationalization, and the outcomes we assessed (Druckman, 2022). Yes, our findings match similar studies in other domains (Flores and Coppock, 2018; Pérez and Tavits, 2022), but the lack of support here for a *cultural* account does not imply full disconfirmation. In fact, we think the more constructive way to view these two accounts is additively, where cultural considerations sometimes condition language effects. Seen this way, we simply failed to find evidence of that conditional effect here, in the realm of COVID-19, based on the treatments and outcomes we employed. Future research should re-consider our findings in light of alternate treatments and outcomes from the domain of COVID-19 (Druckman, 2022). Additionally, scholars might wish to explore the influence of potential moderators of this hypothesized relationship, including the role of trust in government among Latinos and other people of color (Pérez, 2021).

We also bring attention to another implication of our results. Although interview language had systematic effects on opinions in this domain, this pattern was largely specific to reported *levels* of opinion toward COVID-19 health protocols. Yet interview language seemed to have a much harder time *changing* these opinions. Information about COVID-19 is a saturated field, with widely and consistently disseminated messages about the (de)merits of these measures. This suggests an urgent need to imagine additional treatments that might persuade U.S. Latino adults to change their opinions in a domain that is severely affecting their overall well-being.

We conclude by discussing what our results imply about assessments of public opinion toward COVID-19 in linguistically diverse polities. Our findings suggest that interview language provides more than administrative data on survey respondents. Indeed, the tongue one uses to report opinions about COVID-19 is indelibly affected by the tongue one uses to report those attitudes. This means that meaningful characterizations of U.S. opinion about COVID-19 require sizeable samples of respondents who interview in (non-)English languages. While some researchers might balk at the financial costs associated with designing and yielding such samples, it is important to note that in the U.S., an estimated 75% of the Latino population (5 years and older) speaks Spanish at home (Krogstad and Lopez, 2017). With the Latino population in major U.S. states (e.g., California, Texas, and Florida) sometimes approaching nearly half of a state’s total population, an unbiased assessment of U.S. opinion toward COVID-19 demands more effort in removing obstacles to conducting polls in multiple languages—a challenge the U.S. shares with other linguistically diverse polities in Western Europe and across the globe (Pérez and Tavits, 2022).

## Supplemental Material

Supplemental Material - Unexpected, but consistent and pre-registered: Experimental evidence on interview language and Latino views of COVID-19 Click here for additional data file. Supplemental Material for Unexpected, but consistent and pre-registered: Experimental evidence on interview language and Latino views of COVID-19 by Efrén Pérez, Jessica HyunJeong Lee, Ana L Oaxaca CarrascoAna L Oaxaca Carrasco, Cole Matthews, and Madison Ritsema in Research and Politics.

## Notes

1 In all our treatments, the proposed COVID-19 protocols require an object of influence (i.e., *people*). Since *people* is a highly heterogeneous group, we reasoned it would introduce unnecessary noise as respondents imputed their own sense of who *people* are, thus complicating our interpretation of any results. For this reason, we did not include additional two conditions with *people* as the reference category.

2 Prior work shows later-generation Latinos express higher levels of *American* versus *Latino* identity (Citrin and Sears 2014). This does not mean their *Latino* identity is unimportant to them. Instead, Latinos have access to both attachments, whose salience varies by context, including surveys (Pérez, 2016). Crucially, psychologists have detected the effects of cultural norms on bilingual populations like Latinos by using manipulations that are more *and* less involved than ours (e.g., priming norms through images, recall tasks) (Hong et al., 2000; Marian and Neisser, 2000).
