# Supplementary material for: Unexpected, but consistent and pre-registered: Experimental evidence on interview language and Latino views of COVID-19
Source: Research & Politics. 2023 Apr 19;10(2):20531680231168736. doi: 10.1177/20531680231168736 (PMC10116200; doi:10.1177/20531680231168736)
Supplement: Supplemental Material - Unexpected, but consistent and pre-registered: Experimental evidence on interview language and Latino views of COVID-19 [file sj-pdf-1-rap-10.1177_20531680231168736.pdf]

Supplementary Information (SI)
for
"Experimenting with Interview Language and Latino Support for COVID-19 Protocols"

1) SI.1. Sample Composition
2) SI.2. Balance Tests
3) SI.3. Treatments: Wording and Graphics
4) SI.4. Anonymous Pre-Registration for the Project

SI.1. Sample Composition

Table SI.1. Sample Composition

Age (median years)	23 years
U.S.-born (%)	74%
Female (%)	70%
College-educated (%)	31%
N	1,706

SI.2. Balance Tests

The tables below report the balance tests for our sample, corrected for multiple comparisons. Across 12 comparisons, we find that 2 produce a low *p*-value (i.e., less than .05, two-tailed). This is unsurprising given our large sample size and the multiple-comparisons conducted. With a Bonferroni correction, the critical value to surpass is $p<.004$, two-tailed. None of the comparisons involving our pretreament data cross this threshold, further suggesting our experiment is generally balanced across conditions.

Table SI.2. Balance Tests (Corrected for Multiple Comparisons at $p<.004$)

Age (years)	*t*-test		*t*-test		*t*-test
Control	23.963	Control	23.963	Control	23.963
English-Collective	23.256	Spanish-Individual	22.267	Spanish-Collective	20.992
	$p<.581$		$p<.180$		$p<.019$

Female (%)	*t*-test		*t*-test		*t*-test
Control	69.30	Control	69.30	Control	69.30
English-Collective	72.00	Spanish-Individual	65.99	Spanish-Collective	70.45
	$p<.387$		$p<.310$		$p<.719$

Continued below

College (%)	*t*-test		*t*-test		*t*-test
Control	32.10	Control	32.10	Control	32.10
English-Collective	30.12	Spanish-Individual	32.49	Spanish-Collective	27.27
	$p<.533$		$p<.904$		$p<.131$

US-born (%)	*t*-test		*t*-test		*t*-test
Control	76.28	Control	76.28	Control	76.28
English-Collective	77.18	Spanish-Individual	68.78	Spanish-Collective	71.97
	$p<.757$		$p<.016$		$p<.158$

SI.3. Treatments: Wording and Graphics

English (Individualist)

Amid a plateau in COVID-19 vaccinations, the Centers for Disease Control and Prevention (CDC) reconsiders a new mask mandate and stronger efforts to boost vaccination rates among individuals

-Associated Press

Although COVID-19 infections are not rising as rapidly as before, health researchers have observed a plateau in the rate of individuals getting COVID-19 vaccines or booster shots. This means that, although booster vaccinations and mask use are effective at preventing COVID-19 infection, too many new infections are undercutting gains in individual protection against the coronavirus and its variants. In short, not enough is being done to prevent new infections, which foreshadows long personal battles with COVID-19. The worst effects of COVID-19 infections are being felt among unvaccinated individuals, since they are more likely to be hospitalized due to COVID-19 than vaccinated individuals. This trend is illustrated in the figure below.

Figure: Unvaccinated individuals are more likely to be hospitalized due to COVID-19

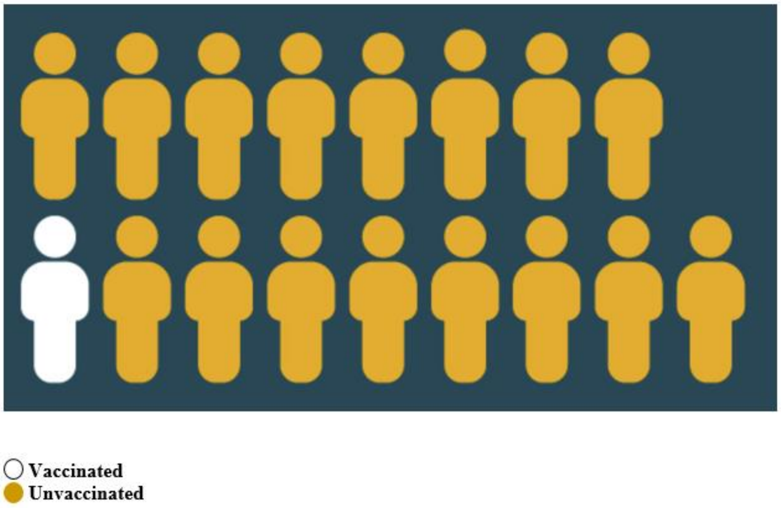

Source: Center for Disease Control and Prevention CDC

At a recent press briefing, a Centers for Disease Control and Prevention (CDC) spokesperson noted that, in light of these new COVID-19 data, the CDC is strongly reconsidering issuing a new national mask mandate and investing in national efforts to fully vaccinate more Americans, including those individuals who have not received a booster shot. “For the sake of each person’s health,” the spokesperson stated, “these new health protocols will require personal sacrifices in order to avoid more individuals being hospitalized and dying. You should do it for yourself. You should do it for your own health.”

English (Collectivist)

Amid a Plateau in COVID Vaccinations, the Centers for Disease Control and Prevention (CDC) Reconsiders a New Mask Mandate and Stronger Efforts to Boost Vaccination Rates in Communities

-Associated Press

Although COVID infections are not rising as rapidly as before, health researchers have observed a plateau in the rate of communities getting COVID-19 vaccination or booster shots. This means that, although booster vaccinations and mask use are effective at preventing COVID-19 infection, too many new infections are undercutting our gains in community protection against the coronavirus and its variants. In short, not enough is being done to prevent new infections, foreshadowing long collective battles with COVID-19. The worst effects of COVID-19 infection are felt in unvaccinated communities, since they are more likely to be hospitalized due to COVID-19 than vaccinated communities.

Figure: Unvaccinated communities are more likely to be hospitalized due to COVID-19

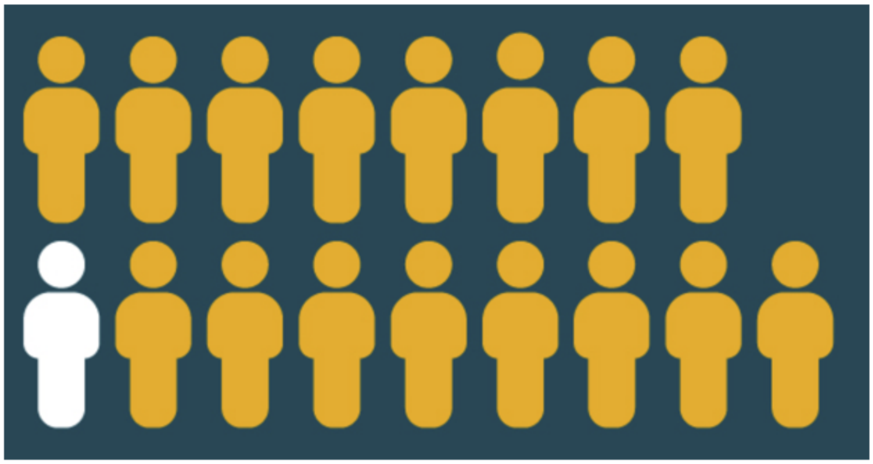

○ **Vaccinated**
● **Unvaccinated**

Source: Center for Disease Control and Prevention

At a press briefing today, a Centers for Disease Control and Prevention (CDC) spokesperson noted that, in light of these new COVID-19 data, the CDC is strongly reconsidering issuing a new national mask mandate and investing in national efforts to fully vaccinate more Americans, including those who have not received a booster shot. "For the sake of community health," the spokesperson stated, "these new public health protocols will require collective sacrifices in order to avoid more hospitalizations and deaths. We should do this for our communities. We should do this for communities' health."

Spanish (Individualist)

En medio de un estancamiento en vacunaciones contra el COVID-19, los Centros para el Control y la Prevención de Enfermedades (CDC) reconsideran un nuevo mandato sobre el uso de mascarillas y mayores esfuerzos para aumentar el porcentaje de vacunación entre individuos

- AP Noticias

Aunque las infecciones de COVID-19 no están aumentando tan rápidamente como antes, los investigadores de salud han observado un estancamiento en el **porcentaje de individuos** que estan recibiendo vacunas y vacunas de refuerzos contra el COVID-19. Esto significa que, aunque las vacunas de refuerzo y el uso de mascarillas son eficaces para prevenir la infección de COVID-19, demasiadas infecciones nuevas están disminuyendo los avances en la protección **individual** contra el coronavirus y sus variantes. En resumen, no se está haciendo lo suficiente para prevenir nuevas infecciones, lo que presagia largas batallas **personales** contra el COVID-19. Los peores efectos de las infecciones por COVID-19 se sienten **entre los individuos** no vacunados, ya que es más probable que sean hospitalizados debido al COVID-19 que los **individuos vacunados**. Esta tendencia se ilustra en la siguiente gráfica.

Gráfica: las personas no vacunadas tienen más probabilidad de ser hospitalizadas debido al Covid-19

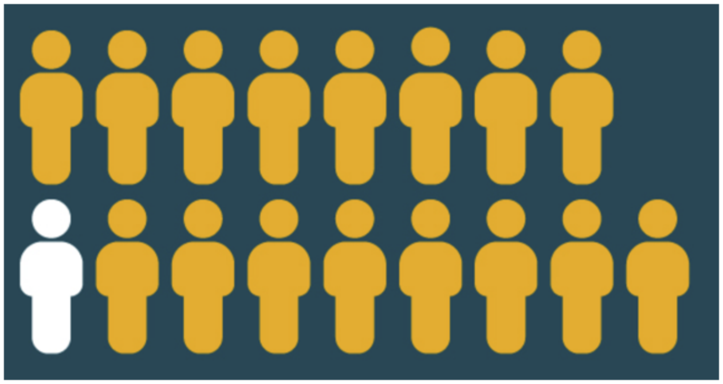

○ **Vacunados**
● **No vacunados**

Fuente: Centros para el Control y la Prevencion de Enfermedades CDC

En una conferencia de prensa reciente, un portavoz de los Centros para el Control y la Prevención de Enfermedades (CDC) señaló que, a la luz de estos nuevos datos de COVID-19, los CDC están reconsiderando seriamente promulgar un nuevo mandato nacional de uso de mascarilla e invertir en esfuerzos nacionales para vacunar por completo a más estadounidenses, incluidos aquellos **individuos** que no han recibido una vacuna de refuerzo. "Por el bien de la salud de **cada persona**", dijo el potavoz, "estos nuevos protocolos de salud requerirán sacrificios **personales** para evitar que más **individuos** sean hospitalizadas y mueran. Deberías hacerlo **por ti mismo**. Deberías hacerlo **por tu propia salud**."

Spanish (Collectivist)

En medio de un estancamiento en vacunaciones contra el COVID-19, los Centros para el Control y la Prevención de Enfermedades (CDC) reconsideran un nuevo mandato sobre el uso de mascarillas y mayores esfuerzos para aumentar el porcentaje de vacunación en las comunidades.

- AP Noticias

Aunque las infecciones de COVID-19 no están aumentando tan rápidamente como antes, los investigadores de salud han observado un estancamiento en el **porcentaje de comunidades** que estan recibiendo vacunas y vacunas de refuerzos contra el COVID-19. Esto significa que, aunque las vacunas de refuerzo y el uso de mascarillas son eficaces para prevenir la infección de COVID-19, demasiadas infecciones nuevas están disminuyendo los avances en la protección **de las comunidades** contra el coronavirus y sus variantes. En resumen, no se está haciendo lo suficiente para prevenir nuevas infecciones, lo que presagia largas batallas **colectivas** contra el COVID-19. Los peores efectos de las infecciones por COVID-19 se sienten **entre comunidades** no vacunadas, ya que es más probable que sean hospitalizadas debido al COVID-19 que las **comunidades vacunadas**. Esta tendencia se ilustra en la siguiente gráfica.

Gráfica: las comunidades no vacunadas tienen más probabilidad de ser hospitalizadas debido al Covid-19

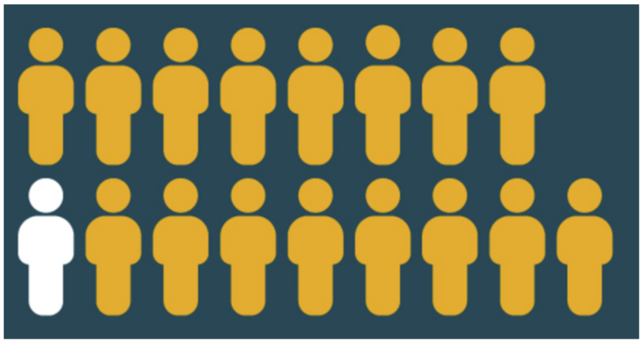

Vacunados
No vacunados

Fuente: Centros para el Control y la Prevencion de Enfermedades CDC

En una conferencia de prensa reciente, un portavoz de los Centros para el Control y la Prevención de Enfermedades (CDC) señaló que, a la luz de estos nuevos datos de COVID-19, los CDC están reconsiderando seriamente promulgar un nuevo mandato nacional de uso de mascarilla e invertir en esfuerzos nacionales para vacunar por completo a más estadounidenses, incluidos aquellos que no han recibido una vacuna de refuerzo. "Por el bien de la salud de **las comunidades**", dijo el potavoz, "estos nuevos protocolos de salud requerirán sacrificios **colectivos** para evitar más hospitalizaciónes y muertes. Deberíamos hacerlo **por nuestras comunidades**. Deberíamos hacerlo **por la salud de las comunidades.**"

SI.4. Anonymous Pre-Registration for the Project

As Predicted: *"Interview Language and Mass Support for COVID-19 Public Health Protocols"* (#97883)

Created: 05/23/2022 06:06 AM (PT)

Author(s)

1) Have any data been collected for this study already?
No, no data have been collected for this study yet.

2) What's the main question being asked or hypothesis being tested in this study?
Does language of interview increase support for public health protocols related to COVID-19? Is the effect of language stronger when the language of interview matches the normative language used by public health messaging? This experiment assigns the language of interview (English/Spanish) to U.S. Latino bilingual adults and crosses this treatment with another manipulation that uses collectivist (individualist) language to persuade public support.

H1) Assignment to interview in Spanish (versus English) increases support for COVID-19 public health protocols.

H2) The effect of interviewing in Spanish (versus English) on increased support for COVID-19 public health protocols is stronger when public messaging contains collectivist (versus individualist) norms.

3) Describe the key dependent variable(s) specifying how they will be measured.
The following four items will be administered pre-treatment, using a 1 to 7 Likert scale (1-strongly disagree/7-strongly agree). The same four items will be re-administered post-treatment. Our primary outcome will be average changes in support for public health protocols related to COVID-19. We plan to create this outcome by 1) subtracting pre-treatment support for an item from post-treatment support for the same item; and 2) taking the average of these differences across all four items.

-The CDC is thinking about issuing a new national mask mandate to slow the transmission of COVID-19. What is your opinion about this proposed measure?
-The CDC is also considering limiting restaurants and stadiums to 50% capacity to slow the transmission of COVID-19. What is your opinion about this proposal?
-The CDC is thinking about making it a requirement to show proof of vaccination for activities in public spaces, such as restaurants and stadiums. What is your opinion about this proposal?
-The CDC is also considering a mandatory quarantine of 7 days for domestic and international travelers if one is infected with COVID-19. What is your opinion about this proposal?

4) How many and which conditions will participants be assigned to?
There are four conditions:

1. English interview, individualist language in public message
2. English interview, collectivist language in public message
3. Spanish interview, individualist language in public message
4. Spanish interview, collectivist language in public message

5) Specify exactly which analyses you will conduct to examine the main question/hypothesis.
We will analyze the data by 1) estimating the effect of each treatment condition (relative to English interview, individualist language) on our primary outcome; 2) estimating the effect of each treatment condition (relative to English interview, individualist language) on our primary outcome in a structural equation modeling framework where our outcome is treated as a latent variable.

6) Describe exactly how outliers will be defined and handled, and your precise rule(s) for excluding observations.
Participants who fail a manipulation check following each condition will be excluded from the analyses.

7) How many observations will be collected or what will determine sample size?
No need to justify decision, but be precise about exactly how the number will be determined.
The plan is to collect N=1600 observations. With N=400 per cell in the experiment, this sample size allows the detection of a small but reliable effect (d~.20, p<.05, two-tailed).

8) Anything else you would like to pre-register?
(e.g., secondary analyses, variables collected for exploratory purposes, unusual analyses planned?)
This pre-registration includes an allowance to estimate the anticipated treatment effects on on levels of our primary outcome (rather than changes).
